# Supplementary material for: Strong light-matter coupling for reduced photon energy losses in organic photovoltaics
Source: Nat Commun. 2019 Aug 16;10:3706. doi: 10.1038/s41467-019-11717-5 (PMC6697723; doi:10.1038/s41467-019-11717-5)
Supplement: Supplementary file 1 — Supplementary Information [file 41467_2019_11717_MOESM1_ESM.pdf]

SUPPLEMENTARY INFORMATION

Strong light-matter coupling for reduced photon energy losses in organic photovoltaics

Vasileios C. Nikolis et al.

SUPPLEMENTARY FIGURES

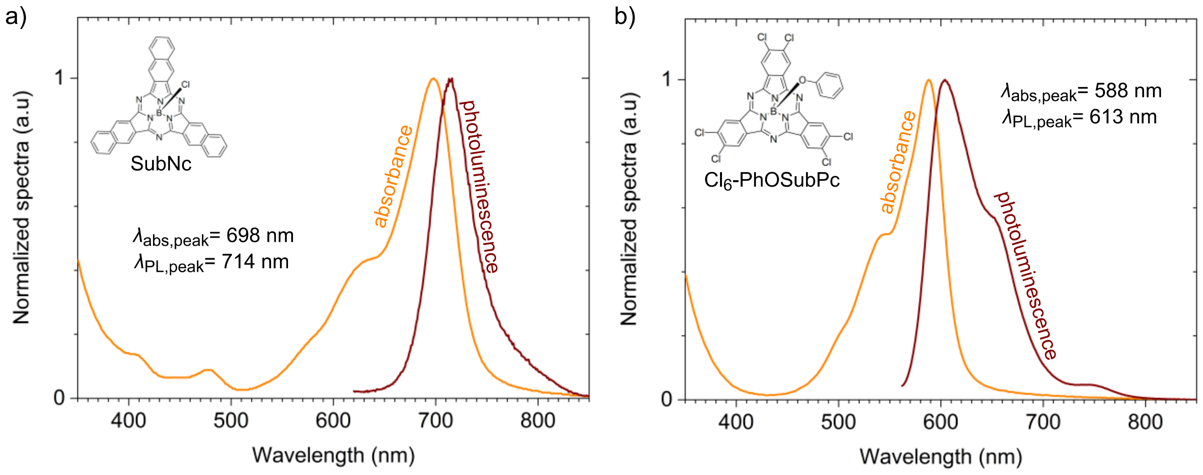

Supplementary Figure 1. Normalized thin film absorbance and photoluminescence spectra for a) chloroboron subnaphthalocyanine (SubNc) film of 12 nm, and b) hexachloro phenoxy subphthalocyanine (Cl_6-PhOSubPc) film of 15 nm. Inset pictures show the molecular structure of each organic absorber. The peak wavelengths for absorbance and photoluminescence are also indicated, and their difference yields a Stokes shift of 16 nm (40 meV) for SubNc, and 25 nm (86 meV) for Cl_6-PhOSubPc.

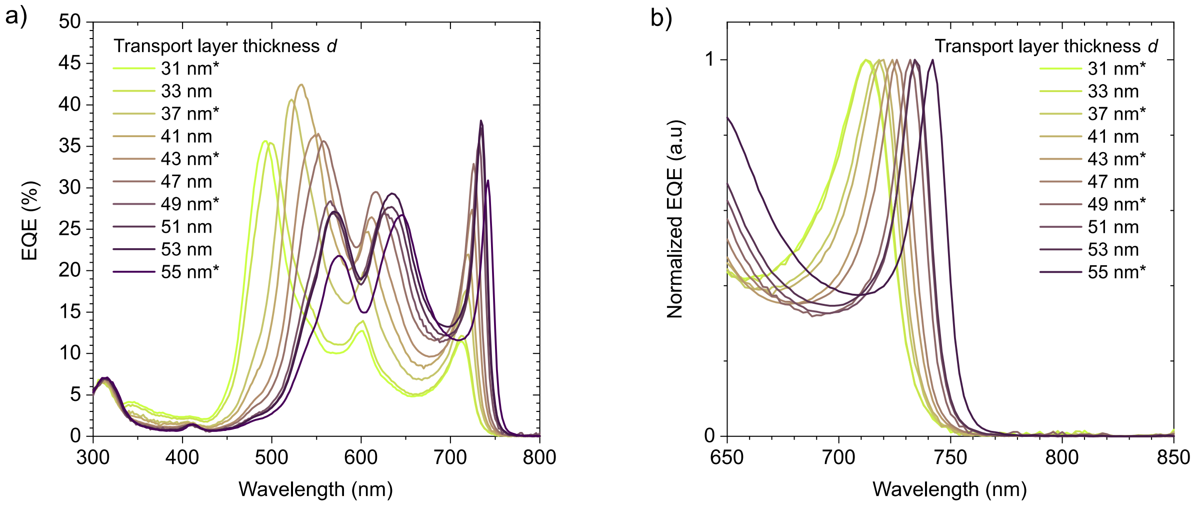

Supplementary Figure 2. EQE spectra of SubNc/Cl_6-PhOSubPc SC-devices with varying *d*. a) External quantum efficiency (EQE) spectra of SubNc/Cl_6-PhOSubPc strong coupling (SC) devices, with varying transport layer thickness *d*. The EQE maxima shown here were used to confirm the simulated device absorption shown in Figure 2b of the main text. The asterisks in the legends denote the samples which were used in the analysis in the main text. b) Normalized EQE spectra of SubNc/Cl_6-PhOSubPc SC-devices focused on the spectral range where the lower polariton (LP) absorbs, showing the redshift of the LP as the device thickness varies.

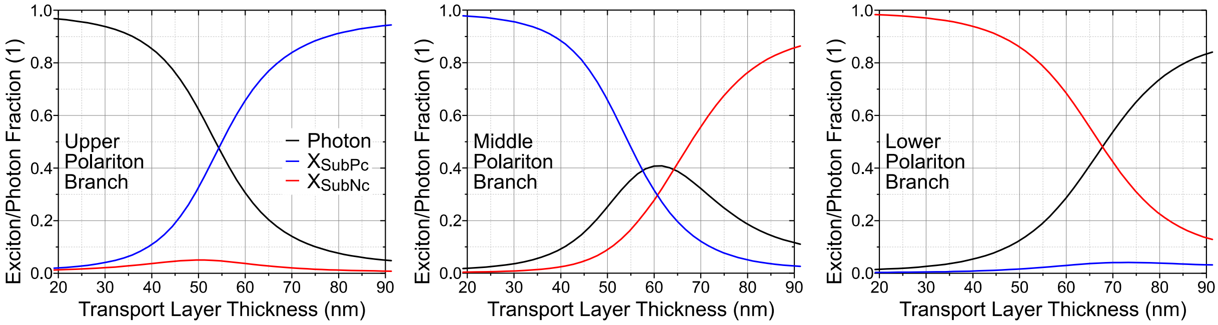

Supplementary Figure 3. Composition of the Upper, Middle and Lower Polaritons in SubNc/Cl_6-PhOSubPc cells. Exciton/photon fraction of upper, middle and lower polariton of SubNc/Cl_6-PhOSubPc SC-devices for varying transport layer thickness.

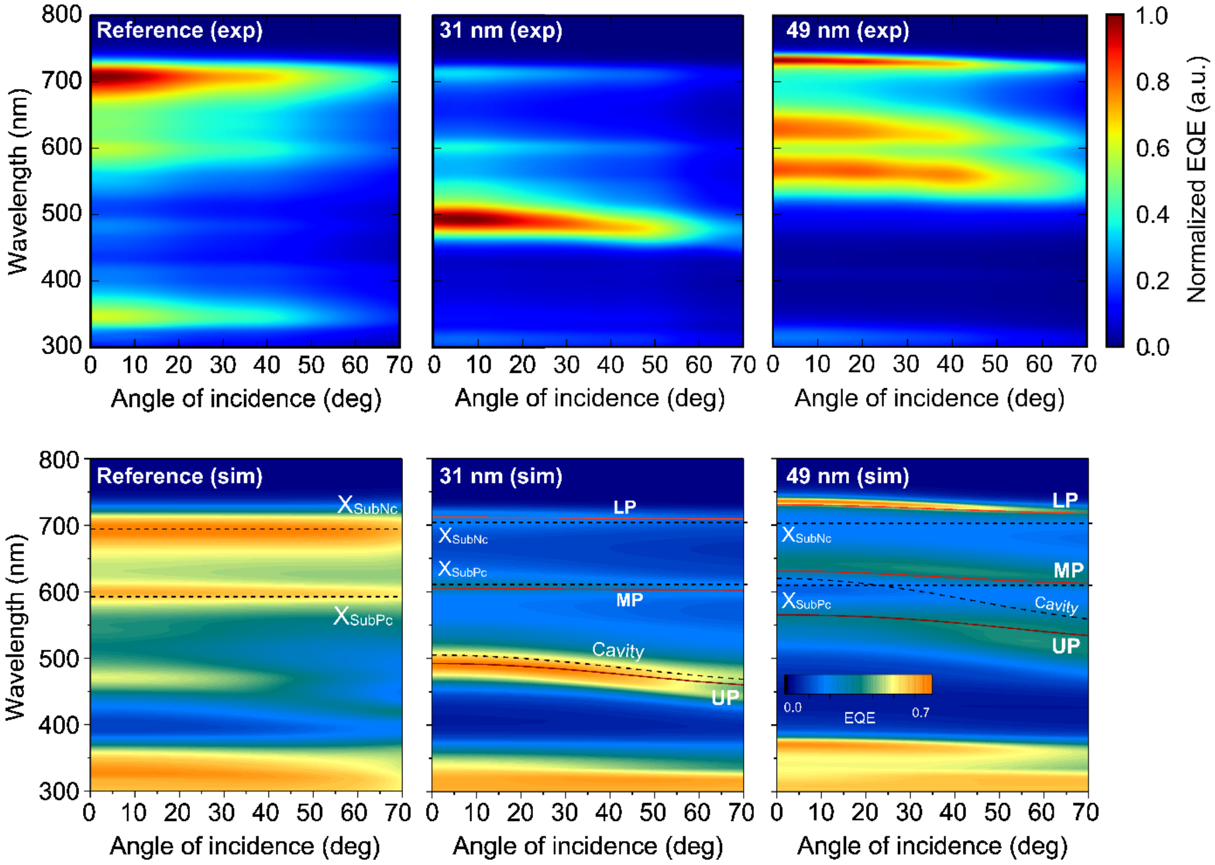

Supplementary Figure 4. Angle-dependence of EQE for the SubNc/Cl_6-PhOSubPc SC-devices. Experimental EQE ("exp", upper panel) and simulated absorption ("sim", lower panel) of the reference SubNc/Cl_6-PhOSubPc device and two SC-devices (d = 31 nm and d = 49 nm), showing the characteristic polariton dispersion, in contrast to the angle-independent reference with ITO.

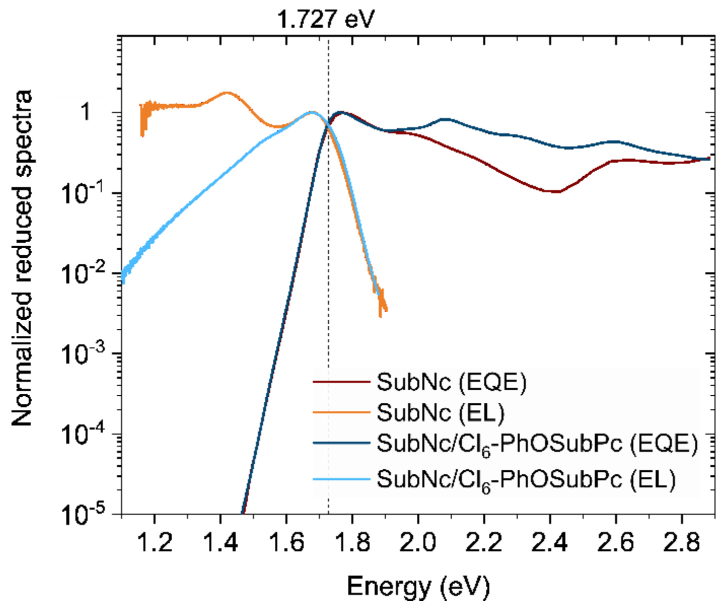

Supplementary Figure 5. EQE and EL spectra of reference SubNc/Cl_6-PhOSubPc and SubNc-only devices. Sensitively measured normalized reduced external quantum efficiency (EQE) and electroluminescence (EL) spectra of a SubNc/Cl_6-PhOSubPc solar cell and a solar cell with only SubNc. The absence of any absorption or emission feature related to CT-states in the SubNc/Cl_6-PhOSubPc device implies a minimal driving force. Therefore, the CT-state energy (E_{CT}) at the SubNc/Cl_6-PhOSubPc interface and the optical gap (E_{opt}) of SubNc coincide and are equal to 1.727 eV.

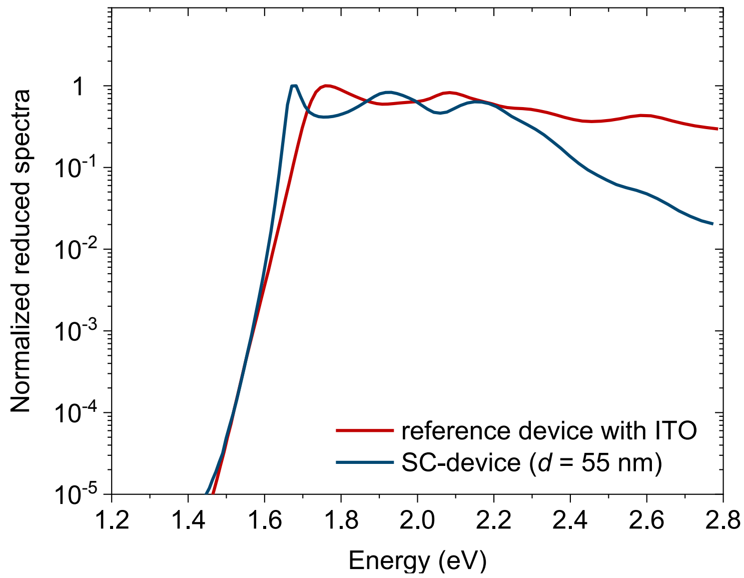

Supplementary Figure 6. Normalized reduced EQE spectra of SubNc/Cl_6-PhOSubPc based SC-device and reference on ITO.

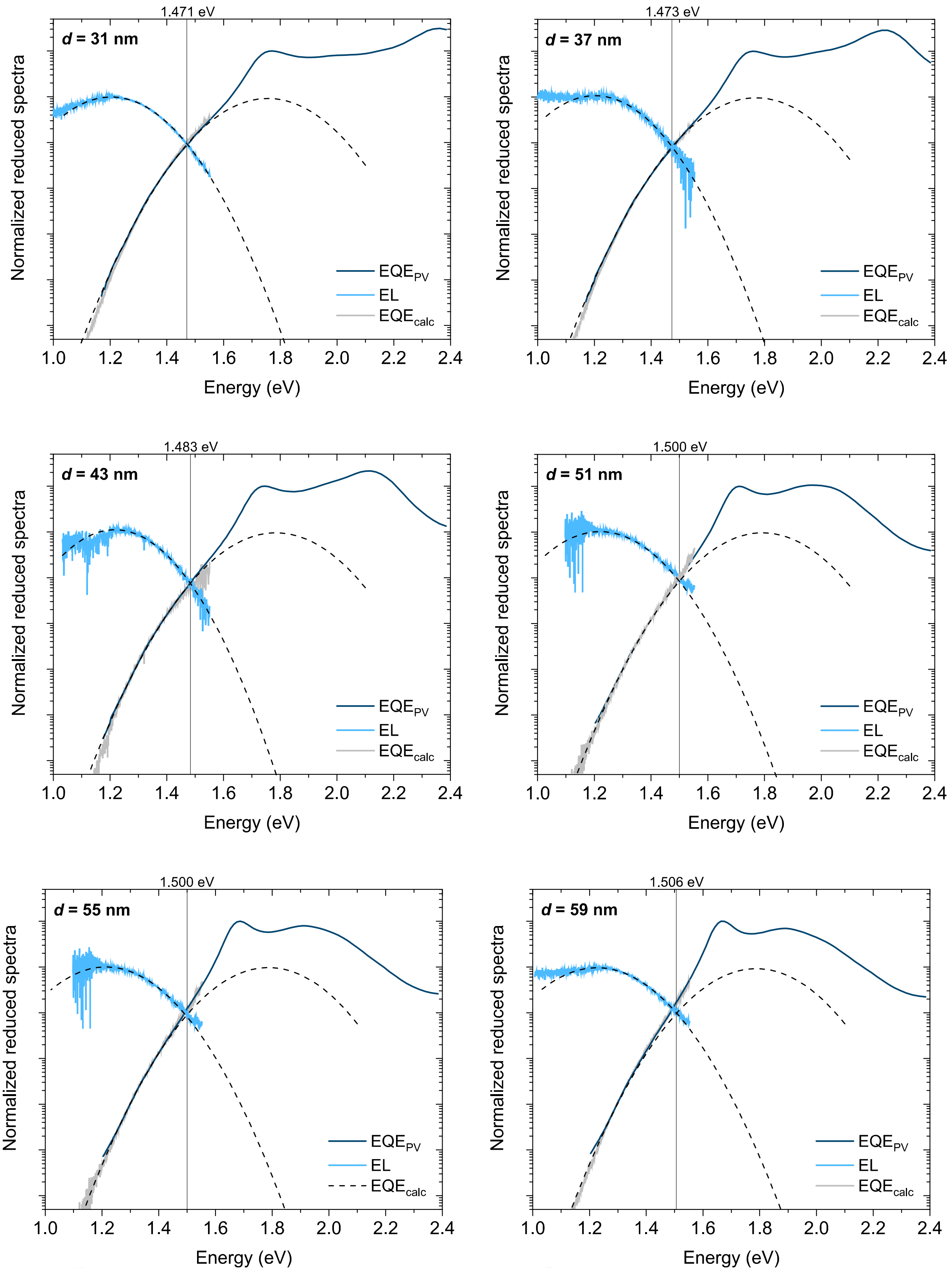

Supplementary Figure 7. Determining the E_{CT} in SubNc/C_{60}-based SC-devices. Normalized reduced external quantum efficiency (EQE, blue) and electroluminescence (EL, cyan) spectra for the SubNc/ C_{60} devices for different transport layer thicknesses *d*. The EL spectra divided by the black body spectrum (EQE$_{calc}$) coincides with the low-energy edge of the EQE spectrum, confirming that the reciprocity between absorption and emission in these devices is valid. Absorption and emission features are fitted with Gaussian fits (dashed lines). The crossing point of the Gaussian fits provide the energy of the CT-state in each case.

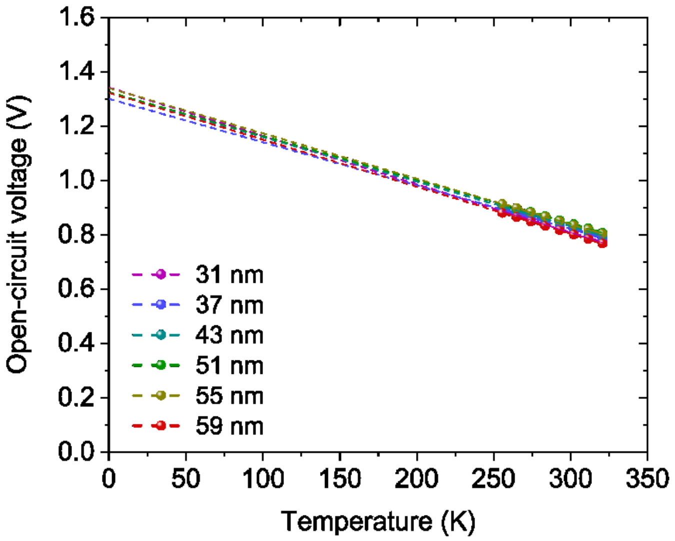

Device	V_{OC} (T = 0 K)	E_{CT} (RT)
31 nm	1.342 V	1.471 V
37 nm	1.300 V	1.473 V
43 nm	1.324 V	1.483 V
51 nm	1.325 V	1.500 V
55 nm	1.340 V	1.500 V
59 nm	1.323 V	1.506 V

Supplementary Figure 8. Temperature-dependent V_{OC} measurements (left) for the SubNc/C_{60} devices for different transport layer thicknesses *d*. The measurements were performed at approximately 1 sun illumination intensity, and at 6 different temperatures from 321 K to 256 K. The colored dashed lines in each case correspond to the linear fits which are used to obtain V_{OC} (T = 0 K), which should equal the energy of the CT-states (E_{CT}) at 0 K.[2] The obtained V_{OC} (T = 0 K) values are summarized for each device in the table (right), including the E_{CT} determined in Figure S7 via EQE and EL measurements at room temperature (RT). V_{OC} (T = 0 K) values are similar for all the devices, between 1.300 V and 1.342 V, showing almost the same variation as the E_{CT} (RT) values (1.471 V to 1.506 V), and confirming that E_{CT} is not affected by strong coupling.

SUPPLEMENTARY TABLES

Supplementary Table 1. Voltage end energy losses for the investigated strongly coupled (SC) SubNc/C_{60} devices with various transport layer thickness *d*. The E_{opt} of the devices corresponds to the peak of the LP branch $\lambda_{peak,LP}$. E_{CT} is determined as the crossing point between appropriately normalized reduced EQE and EL spectra (Figure S6), and found to be approximately the same for the investigated devices. This implies that the driving force ($E_{opt} - E_{CT}$) is reduced via SC in the devices, together with the total voltage losses ($E_{opt} - qV_{OC}$). ΔV_{rad} and ΔV_{nonrad} correspond to the voltage losses related to radiative and nonradiative losses respectively. The calculation of V_{rad} and ΔV_{nonrad} is described in Supplementary Note 1.

d (nm)	$\lambda_{peak,LP}$ (nm)	E_{opt} [a] (eV)	E_{CT} (eV)	V_{OC} (V)	V_{rad} (V)	$E_{opt} - qV_{OC}$ (eV)	$E_{opt} - E_{CT}$ (eV)	ΔV_{rad} [b] (V)	ΔV_{nonrad} [c] (V)
31	700	1.770	1.471	0.790	1.179	0.980	0.299	0.292	0.389
37	705	1.759	1.473	0.787	1.179	0.972	0.286	0.294	0.392
43	710	1.746	1.483	0.800	1.178	0.946	0.263	0.305	0.378
51	725	1.711	1.500	0.799	1.175	0.912	0.211	0.325	0.376
55	735	1.689	1.500	0.802	1.175	0.887	0.189	0.325	0.373
59	743	1.665	1.506	0.791	1.171	0.874	0.159	0.335	0.380

[a] obtained as the peak of the lower polariton branch as $E_{opt} = 1240/\lambda_{peak,LP}$

[b] $\Delta V_{rad} = E_{CT}/q - V_{rad}$

[c] $\Delta V_{nonrad} = V_{rad} - V_{OC}$

SUPPLEMENTARY NOTES

Supplementary Note 1: Calculation of V_{rad} and ΔV_{nonrad}

In the absence of any nonradiative decay, the upper limit for V_{OC} (V_{rad}) is given by:[1]

$$V_{rad} = \frac{k_B T}{q} ln\left(\frac{J_{SC}}{J_0^r}\right) \tag{S1}$$

where J_{SC} is the solar cell's short-circuit current density, here obtained by integrating the product of the device's EQE spectrum and the solar AM1.5G spectrum, J_0^r is the radiative limit of the dark current obtained by integrating the product of the device's EQE spectrum and the black body spectrum at room temperature, k_B is the Boltzmann's constant, and T is the temperature (T=294 K was used in our calculations of V_{rad}). The difference between V_{rad} and V_{OC} refer to the voltage losses occurring nonradiatively (ΔV_{nonrad}):

$$\Delta V_{non-rad} = V_{rad} - V_{OC} \tag{S2}$$

Supplementary Note 2: Dependence of V_{OC} on the steepening of the absorption edge

According to Shockley-Queisser theory, an ideal solar cell in thermodynamic equilibrium absorbs solar radiation φ_{sun} and emits black body radiation φ_{BB}. The absorbed and emitted photon fluxes depend on the absorptance $a(E)$ and the internal quantum efficiency $IQE(E)$ which determine the short-circuit current density:

$$J_{SC} = q\int_0^\infty a(E)IQE(E)\varphi_{sun}(E)dE \tag{S3}$$

where q is the elementary charge and E the photon energy.

Neglecting recombination occurring nonradiatively, an upper limit for open-circuit voltage, namely the radiative open-circuit voltage V_{rad}, can be determined using equation S1. The minimum reverse dark current, J_0^r, also relates to $a(E)$ and $IQE(E)$ by:

$$J_0^r = q\int_0^\infty a(E)IQE(E)\varphi_{BB}(E)dE \tag{S4}$$

where the spectral dependence of the black body radiation is given by:

$$\varphi_{BB} = \frac{2\pi E^2}{h^3c^2}\frac{1}{[\exp(E/kT)-1]} \tag{S5}$$

Band tailing ($E_U > 0$) increases the solar cell's absorption and emission. On one hand, the slight absorption broadening will lead to a slight increase in J_{SC}. On the other hand, J_0^r increases exponentially when $E_U > kT$, due to the exponential dependence of φ_{BB} on E (Equations S4 and S5). Thus, there is a threshold E_U value at k_BT, where we observe two regimes:[3]

1. for $E_U > k_BT$, V_{rad} decreases rapidly due to the exponential increase in the dark current J_0^r. A reduction of E_U in this regime would lead to a significantly increased V_{OC}.

2. for $E_U < k_BT$, J_0^r is not significantly affected, and V_{rad} increases only very slightly due to the slight increase in J_{SC}.

Our measurements were performed at room temperature (T = 298 K), where k_BT is equal to 25.8 meV. For our reference device, E_U is already at 22.4 meV, since SubNc exhibits in general a very steep absorption edge, and by employing strong coupling, we reduce E_U to 15.6 meV in the best case. Thus, it is clear that the whole E_U optimization occurs in the '$E_U < k_BT$' regime for our samples, where V_{OC} is expected to be benefited but only slightly. Based on the model of Jean et al. for disordered semiconductors[3], we estimate that the reduction of E_U from 22.4 meV to 15.6 meV should lead to a voltage increase of approximately 40 mV. Our calculations for the V_{rad} of the investigated devices lead to a 25 mV increase (see Supplementary Table 1), being in the same range.

For the real V_{OC} of our devices we have to consider losses due to nonradiative recombination, charge transport and collection losses, as well as optical losses (due to parasitic absorption and reflection) which can dissipate this predicted marginal gain in voltage and lead to a seemingly non-optimized photovoltage.

SUPPLEMENTARY REFERENCES

1. Rau, U. Reciprocity relation between photovoltaic quantum efficiency and electroluminescent emission of solar cells. *Phys. Rev. B* **76,** 85303 (2007).

2. Vandewal, K., Tvingstedt, K., Gadisa, A., Inganäs, O. & Manca, J. V. Relating the open-circuit voltage to interface molecular properties of donor:acceptor bulk heterojunction solar cells. *Phys. Rev. B* **81,** 1–8 (2010).

3. Jean, J. *et al.* Radiative Efficiency Limit with Band Tailing Exceeds 30% for Quantum Dot Solar Cells. *ACS Energy Lett.* **2,** 2616–2624 (2017).
